# Metabolic Syndrome: Past, Present and Future

**DOI:** 10.3390/nu12113501

**Published:** 2020-11-14

**Authors:** Isabelle Lemieux, Jean-Pierre Després

**Affiliations:** 1 Centre de recherche de l’Institut universitaire de cardiologie et de pneumologie de Québec—Université Laval, Québec, QC G1V 4G5, Canada; jean-pierre.despres.ciussscn@ssss.gouv.qc.ca; 2 Department of Kinesiology, Faculty of Medicine, Université Laval, Québec, QC G1V 0A6, Canada; 3 VITAM—Centre de recherche en santé durable, CIUSSS de la Capitale-Nationale, Québec, QC G1J 0A4, Canada

## 1. Syndrome X: A Tribute to a Pioneer, Gerald M. Reaven

Most clinicians and health professionals have heard or read about metabolic syndrome. For instance, as of October 2020, entering “metabolic syndrome” in a PubMed search generated more than 57,000 publications since the introduction of the concept by Grundy and colleagues in 2001 [1]. Although many health professionals are familiar with the five criteria proposed by the National Cholesterol Education Program-Adult Treatment Panel III for its diagnosis (waist circumference, triglycerides, high-density lipoprotein (HDL) cholesterol, blood pressure and glucose), how these variables were selected and the rationale used for the identification of cut-offs remain unclear for many people. In addition, the conceptual definition of metabolic syndrome is often confused with the tools (the five criteria) that have been proposed to make its diagnosis [2,3].

In the seminal paper of his American Diabetes Association 1988 Banting award lecture, Reaven put forward the notion that insulin resistance was not only a fundamental defect increasing the risk of type 2 diabetes, but he also proposed that it was a prevalent cause of cardiovascular disease [4]. The latter point was a paradigm shift as cardiovascular medicine had, at that time, a legitimate focus on cholesterol in risk assessment and management. Reaven was therefore the first to propose that insulin resistance was a central component of a cluster of abnormalities which included hyperinsulinemia, dysglycemia, high triglycerides, low HDL cholesterol and elevated blood pressure. Under his theory, this constellation of abnormalities would not only increase the risk of type 2 diabetes but would also be a complex risk factor for cardiovascular outcomes, even in the absence of type 2 diabetes. Reaven initially referred to this condition as syndrome X. However, as there is also a syndrome X in cardiology [5,6] and because insulin resistance is a core component of Reaven’s syndrome, insulin resistance syndrome was a term that then gained popularity in the literature [7,8].

As measuring insulin resistance or circulating insulin levels was not considered as feasible on a large scale in clinical practice, a group of experts then examined whether it could be possible to identify insulin-resistant individuals with common clinical tools widely used in primary care [1]. Because of the strong link between abdominal obesity and insulin resistance, the panel thus agreed on the use of waist circumference as a crude index of abdominal adiposity and then proposed sex-specific waist cut-off values [1]. However, these waist circumference thresholds were based on the relationship between waist circumference and body mass index (BMI) values defining obesity (men: 102 cm = 30 kg/m^2^ and women: 88 cm = 30 kg/m^2^) [9]. Thus, waist circumference thresholds were simply determined from BMI values defining obesity and, most importantly, were not based on clinical outcomes. In addition, because waist circumference and BMI are correlated [10], an elevated waist girth, observed in isolation, cannot properly assess abdominal fat accumulation [11]. For instance, a waist circumference of 104 cm in a middle-aged man with a BMI of 26 kg/m^2^ is not the same adiposity phenotype as an age-matched man with the same waist girth but with a BMI of 32 kg/m^2^. In this specific example, the man with a BMI of 26 kg/m^2^ is clearly abdominally obese (high-risk form of obesity) whereas the man with a BMI of 32 kg/m^2^ is mostly characterized by overall obesity. This is why a recent consensus paper on the use of waist circumference in clinical practice has proposed that waist circumference should not be measured as a single adiposity index but rather interpreted along with the BMI in order to properly discriminate abdominally obese (higher risk) from overall obese (lower risk) persons [11]. 

Regarding simple metabolic markers of insulin resistance and other indices of metabolic syndrome, triglycerides, HDL cholesterol levels and blood glucose are easily obtained from routine clinical biochemistry laboratories, whereas blood pressure is measured in primary care. On that basis, it was proposed that individuals showing any combination of any three out of these five simple clinical criteria were likely to be characterized by insulin resistance. Prospective analyses have also shown that any combination of these factors was predictive of an increased risk of both type 2 diabetes and cardiovascular disease [12,13,14,15,16,17]. 

As it had also been suggested that the waist cut-offs initially proposed were probably too high, their values were thereafter lowered in harmonized criteria proposed by other organizations [18]. Studies have shown that subgroups of individuals meeting or not meeting the clinical criteria of metabolic syndrome (harmonized or not) were quite distinct from each other in terms of risk of type 2 diabetes and cardiovascular disease [12,13,14,15,16,17]. Of course, using different waist circumference cut-off values generated different prevalence values but the subgroups identified were nevertheless found to show different levels of risk.

## 2. From Syndrome X, Insulin Resistance/Metabolic Syndrome to Excess Visceral Adiposity

Because Reaven could find nonobese individuals with insulin resistance and individuals with obesity who were insulin sensitive, he did not include obesity in his initial definition of syndrome X. In that regard, early imaging studies measuring adiposity with the use of computed tomography initially conducted by Matsuzawa and colleagues and by ourselves suggested that there was a remarkable heterogeneity in abdominal fat accumulation (visceral vs. subcutaneous) [19,20]. Additionally, subgroup analyses revealed that there was substantial variation in glucose tolerance as well as in plasma insulin and lipoprotein levels among equally overweight or obese individuals characterized by low or high levels of visceral adipose tissue [21,22,23,24]. Since then, many large cardiometabolic imaging studies have shown that an excess accumulation of visceral adipose tissue (and not of subcutaneous fat) was a key correlate of the features of insulin resistance, explaining why Reaven could not find a robust association between total body fatness and his syndrome X: it was all about body fat distribution [2,3,25,26,27,28,29,30,31].

## 3. Liver Fat: A Key Partner in Crime in Visceral Obesity

More recently, with the availability of magnetic resonance spectroscopy, it has become possible to noninvasively measure with great accuracy liver fat accumulation. With the use of this technique, excess liver fat has been found to be associated with essentially the same clustering metabolic abnormalities as those observed in visceral obesity [32,33,34]. It is important, however, to point out that excess liver fat in isolation (in the absence of excess visceral adipose tissue) is a relatively rare phenomenon as its most frequent form is accompanied by high levels of visceral adipose tissue [35,36,37]. Thus, it has recently become obvious that the most dangerous adiposity phenotype includes excessive amounts of both visceral adipose tissue and liver fat, which is by far the most prevalent form of insulin resistance or metabolic syndrome [31]. On that basis, we have proposed that the clustering abnormalities of excess visceral adiposity/liver fat for which insulin resistance is a key feature should be called Reaven syndrome [3,38].

## 4. This Issue

Despite the progress made in our understanding of the constellation of atherogenic and diabetogenic abnormalities found in the subgroup of individuals with excess levels of visceral adipose tissue and liver fat, many questions remain regarding their etiology and the most efficient approaches to prevent or to manage it. 

Some of these questions are examined in this special issue of *Nutrients*. The reader will find a mix of narrative reviews and communications written by well-published investigators, top international experts in the field. We are very grateful to these experts who have agreed to contribute to this issue [39,40,41,42,43,44,45,46,47,48,49]. Original papers that are relevant to our theme are also included [50,51,52,53,54,55,56,57,58,59].

As expected from the topics covered in *Nutrients*, this issue deals mostly with dietary factors, although some other important lifestyle features, such as physical activity/exercise and sleeping habits, are addressed. Both individual- and population-based solutions are discussed. For instance, the link between dietary fat as well as dietary fructose and sugar-sweetened beverages and some chronic diseases is reviewed. Considering the importance of physical activity/exercise and cardiorespiratory fitness in the prevention and treatment of features of metabolic syndrome, some papers review the literature relevant to these topics. Moreover, other papers deal with the assessment of metabolic syndrome in various age and ethnic groups. Finally, other highly relevant themes are explored, such as sleep habits, sleep apnea and the development of metabolic syndrome and lifestyle habits, the endocannabinoidome and features of metabolic syndrome.

## 5. The Future

Of course, it was not possible to cover all topics relevant to the assessment, prevention and management of such a complex modifiable risk factor which results from the interaction of genetic and environmental/lifestyle factors. The established relationship between the presence of metabolic syndrome and the development of type 2 diabetes and cardiovascular disease has been amply demonstrated, but the interest around metabolic syndrome and visceral obesity is renewed as it has also been related to other chronic diseases, such as brain health and some types of cancer [60,61]. Numerous studies are currently under way to confirm these relationships, to elucidate the underlying mechanisms or even to examine whether lifestyle intervention habits could prevent these diseases and improve their treatment. As we are going through a major epidemic of chronic lifestyle diseases, metabolic syndrome, although criticized as a concept, has been helpful as a screening approach to better identify a subgroup of high-risk individuals who would benefit from clinical and population-based approaches targeting their lifestyle habits. Finally, with the relatively new concept of precision lifestyle medicine, which consists of simultaneously taking into account the individual’s genetic profile as well as his/her living environments and lifestyle habits [62], we propose that the multiplex modifiable risk factor that represents metabolic syndrome will require concerted efforts between clinical approaches and public health solutions if we want to reduce the burden associated with this condition. We hope that the content of this Special Issue will be found useful.
